# Secretan’s Syndrome: It Is Time to Talk About It

**DOI:** 10.7759/cureus.62580

**Published:** 2024-06-18

**Authors:** Achraf Tebbaa El Hassali, Mohammed Barrached, Adnane Lachkar, Najib Abdeljaouad, Hicham Yacoubi

**Affiliations:** 1 Department of Traumatology and Orthopedics, Mohamed I University and Mohammed VI University Hospital, Oujda, MAR

**Keywords:** psychotherapy, factitious, hand, edema, secretan's syndrome

## Abstract

Secretan’s syndrome is a rare condition; the exact etiology remains unclear. It has no specific treatment and the care must be multidisciplinary and personalized. We report a case of a young female patient who presented with a unilateral and painful swelling of the dorsum of the right hand. The diagnosis and treatment of this patient were challenging.

## Introduction

Secretan’s syndrome, characterized by the appearance of hard edema, sometimes with cyanosis, on the dorsum of one or both hands, was first described in 1901 by the Swiss doctor Henri-François Secretan (1856-1916) [1,2]. The exact etiology of this syndrome remains unclear. The initial location at the dorsum of the hand has been extended to other locations including the foot or the ankle. It is considered a subtype of Munchausen syndrome also called “pathomimia” or “factitious disorder” [2,3].

We report a case of Secretan’s syndrome without triggering factor in a 17-year-old patient, discussing it with data from recent literature. The interest of our case lies in its rarity and in its psychosomatic involvement, which constitutes a real diagnostic and therapeutic challenge.

## Case presentation

This is a case of a 17-year-old female patient, a high school student, with no notable personal or family pathological and psychiatric history. She has also no history of taking medication or drug abuse.

She consulted for a unilateral and painful swelling of the dorsum of the right hand, which had appeared gradually over the past 20 days without any notion of trauma, triggering factor, or other associated signs.

On clinical examination, the patient was conscious, stable neurologically and hemodynamically (blood pressure: 130/80 mmHg, heart rate: 79 beat/min), had a respiratory rate of 20 cycles/min, her temperature was 37°C, and her body mass index was 20.5 kg/m^2^. Pitting edema was present at the dorsum of her right hand going up to the wrist, with pain on palpation; the hand was cold with no inflammatory signs. The pain reported by the patient is intermittent of moderate intensity (VAS pain 5/10), limited to the right hand, mechanical in nature, and exacerbated by palpation and mobilization.

**Figure 1:**
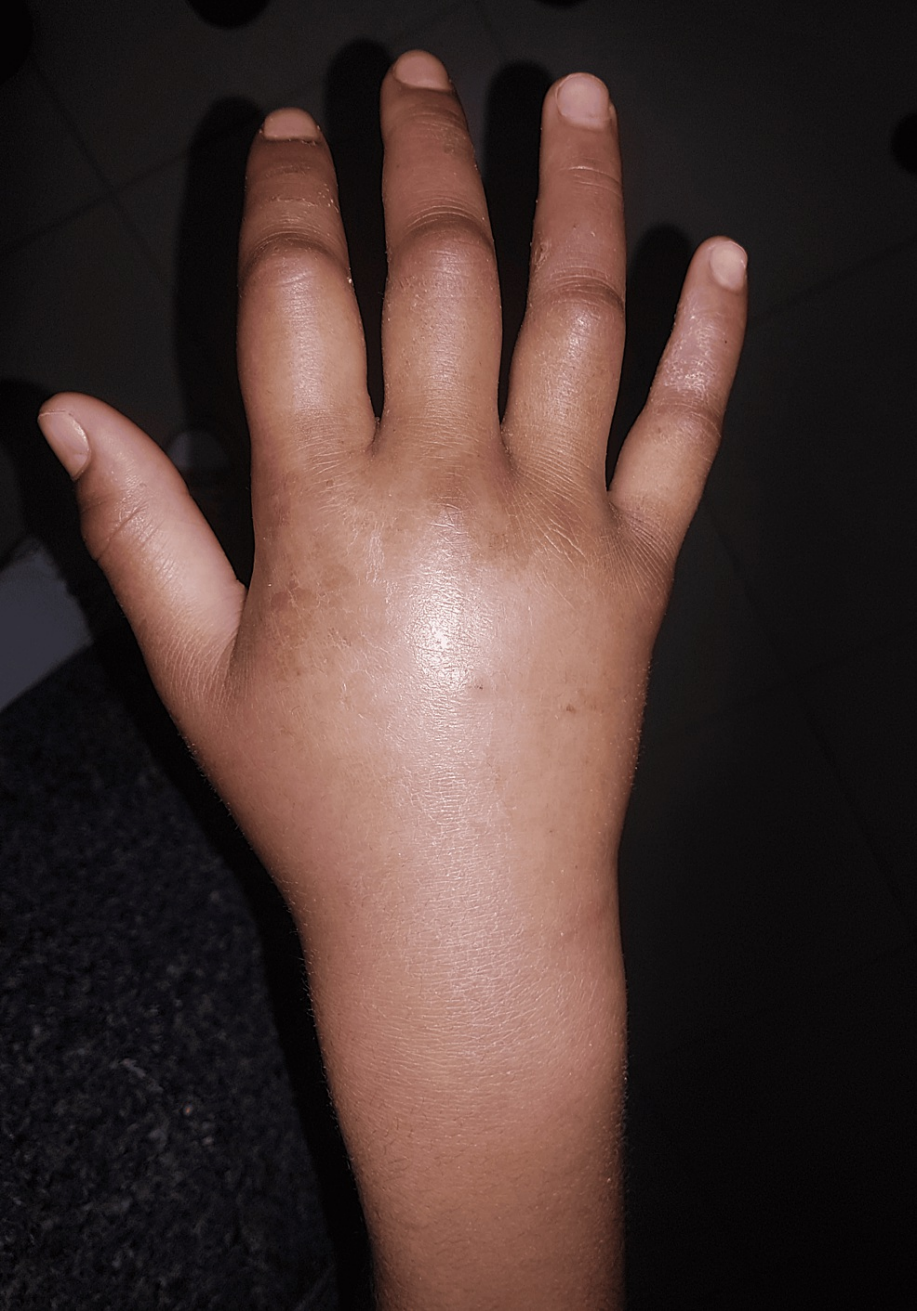
An image of the dorsum of the hand showing swelling

**Figure 2:**
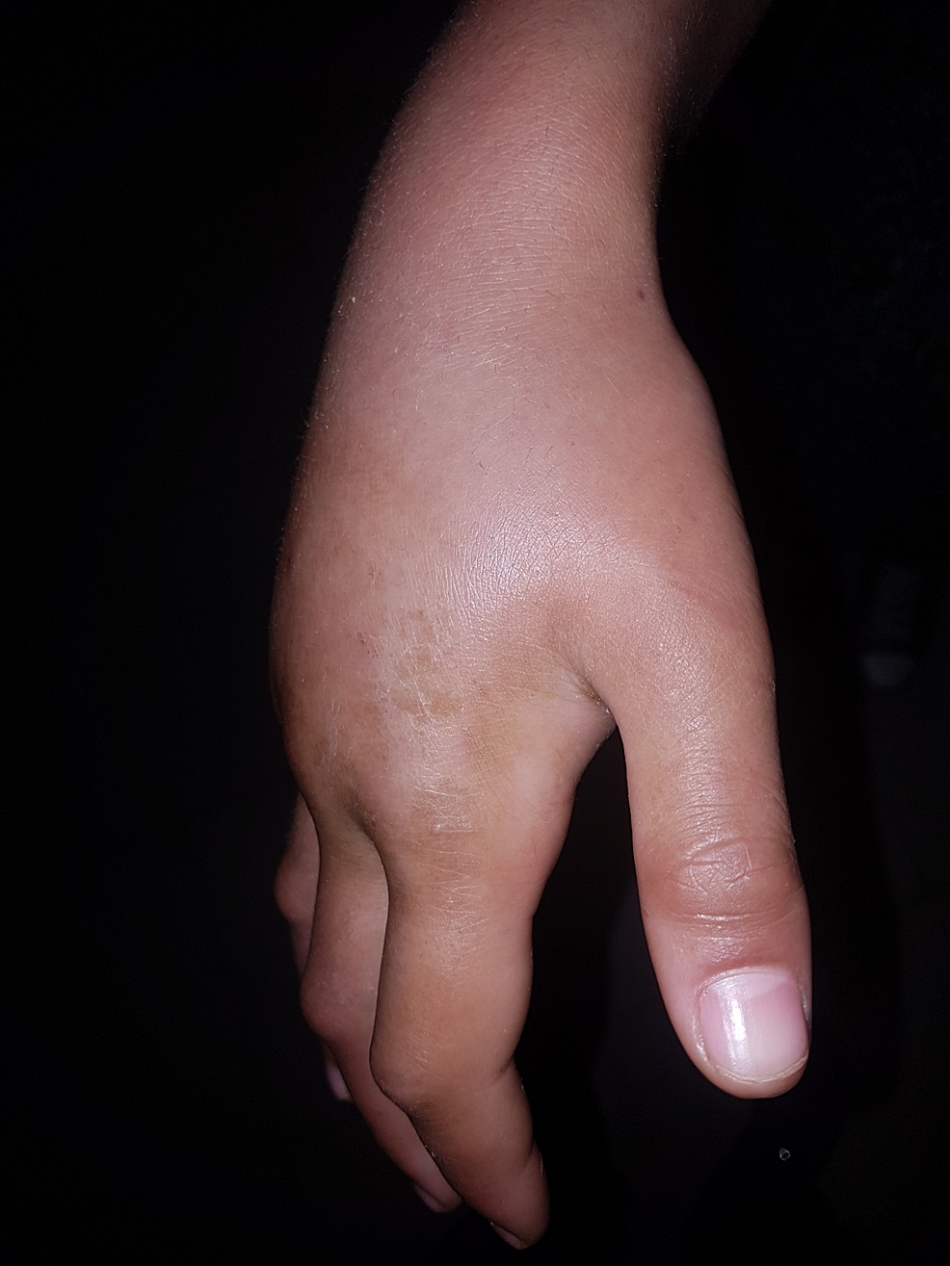
A lateral image of the hand showing swelling

Active mobility is refused by the patient given the pain and passive flexion of the fingers are limited. Furthermore, the vascular and nervous examinations were normal. The examination of the opposite limb as well as the rest of the musculoskeletal system was without abnormality. The clinical appearance of the hand is represented in Figures 1, 2.

She had an X-ray of the right hand on anteroposterior (AP) and lateral views that were unremarkable. The complete biological assessment carried out was normal as presented in Table 1; the patient also had an electrocardiogram (ECG) that showed no abnormalities.

**Table 1:** The biological assessment of the patient

Parameter	Patient’s values	Normal values
C-reactive protein	1.68 mg/L	<6 mg/L
Creatinine	7.20 mg/L	5.7-11.1 mg/L
Urea	0.28 g/L	0.15-0.39 g/L
White blood cells	7500/μL	4000-10.000/μL
Sodium	140 mmol/L	135-145 mmol/L
Potassium	3.9 mmol/L	3.5-4.5 mmol/L
Calcium	2.30 mmol/L	2.25-2.62 mmol/L
Glycemia	0.80 g/L	0.70-0.95 g/L
Aspartate aminotransferase	15 IU/L	5-34 IU/L
Alanine aminotransferase	30 IU/L	0-55 IU/L
Gamma-glutamyl transferase	25 IU/L	9-36 IU/L
Alkaline phosphatase	99 IU/L	40-150 IU/L
Lactate dehydrogenase	159 IU/L	125-220 IU/L
Hemoglobin	13 g/dL	12-16 g/dL
Platelet count	360000/μL	150 000-400 000/μL
Protein	76 g/L	65-80 g/L
Albumin	42 g/L	35-50 g/L
Prothrombin	92%	70-100%

Ultrasound of the right hand suggested phlegmonous infiltration. Magnetic resonance imaging (MRI) showed no abnormalities other than soft tissue edema of the right hand.

The patient was put on paracetamol 3 g/day. She took the treatment only if she experienced pain for four days (one to three doses per day) then she stopped it. The course after discharge (7 days later) from the hospital was marked by spontaneous resolution of the edema after five days, without any surgical treatment. This was predicted by the patient.

A psychiatric interview (suggested to ease her anxiety) with the patient was able to individualize some borderline personality traits such as impulsivity, chronic feeling of emptiness, emotional and relational instability, and an in-depth understanding of medical knowledge with a reluctance toward the causes of her illness. In fact, she knows the anatomy of the hand well. The patient reports also having read several scientific articles and books about factitious disorders. She also knows a former patient that we treated in our department and therefore knows the care circuit well.

Cognitive behavioral therapy was indicated and followed regularly by the patient after the diagnosis of Secretan’s syndrome was announced. The cognitive behavioral therapy sessions aimed to learn to regulate emotions, tolerate distress, and manage interpersonal conflicts differently than with a self-inflicted injury, managing anxiety and validating feelings of suffering and finally expression of emotions and self-affirmation.

After eight sessions, she was able to confirm the self-inflicted nature of the edema without explaining the mechanism. She says that it was a kind of call for help and I quote "I was in pain and no one realized, I did this to encourage my loved ones to help me to stop suffering." The patient's follow-up for 12 months did not mention any recurrence of physical or psychological symptoms or medication intake.

## Discussion

Secretan’s syndrome has been described through several reported cases of Swiss tunnel-boring machine workers who presented with bone fractures and persistent hand lesions that could not be explained by the minimal trauma suffered [2,3]. This clinical description was subsequently broadened to include other locations, notably the lower limbs. Three clinical forms that currently exist are the benign form, the hyperplastic form, and the mixed form. In the benign type, soft tissue infiltration occurs and improves well with psychiatric care. The hyperplastic type occurs after repeated trauma and can be complicated by hematoma or fibrosis and, therefore, requires medical-surgical care associated with psychological care, and in the mixed type the combination of the two forms is found [3,4]. The exact cause of this disorder remains poorly understood but factitious trauma and pathomimia are often suspected [4].

A similarity with self-mutilation and Munchausen syndrome is reported in the literature. Secretan’s syndrome is included in the DSM (the Diagnostic and Statistical Manual of Mental Disorders) as “a factitious disorder with physical signs and symptoms” [4,5].

The factitious nature of hand edema can be suspected in the presence of several of the following arguments: an atypical clinical presentation, personal or family psychiatric history, an unusual clinical course, inconclusive results of blood and radiological tests, a worsening or spectacular improvement under treatment or upon discharge from hospitalization, a prediction of improvement in symptoms, repeated medical consultations with excessive demand for explorations and invasive procedures, etc. [5-8].

Caregivers must be attentive to the symptoms reported and must offer psychological treatment each time an artificial origin is suspected [9-11].

## Conclusions

Secretan’s syndrome is a rare and underdiagnosed clinical entity. Considered as an elimination diagnosis, the medical approach must be systematic and meticulous. No specific treatment is indicated. Care must be multidisciplinary and personalized.
